# Genome sequence of Soos: a siphovirus of the CP cluster infecting *Gordonia rubripertincta*  

**DOI:** 10.1128/mra.01204-23

**Published:** 2024-03-25

**Authors:** Reese M. Adams, Holly A. Britton, Emily D. Bruce, Yucita De La Paz, Emily N. Kratz, Emma J. Pfeifer, Daisy E. Priddy, Brooklyn I. Schotter, Wyatt A. Stuffle, Jordyn Wagner, Meredith R. Weiss, Danielle K. Watt, Pamela L. Connerly, Elizabeth E. Rueschhoff

**Affiliations:** 1 School of Natural Sciences, Indiana University Southeast, New Albany, Indiana, USA; Loyola University Chicago, Chicago, Illinois, USA

**Keywords:** bacteriophage, genome, siphovirus

## Abstract

Novel actinobacteriophage Soos was isolated and purified from Southern Indiana soil using host *Gordonia rubripertincta* NRRL B-16540. Sequencing revealed a 57,509 bp circularly permuted genome encoding 87 predicted protein-coding genes. Soos is only the third phage in cluster CP, along with phages Clawz and Sting.

## ANNOUNCEMENT

*Gordonia* species are commonly found in soil and water and have occasionally caused illness (1). To date, over 720 *Gordonia* phages have been sequenced (2).

**Fig 1:**
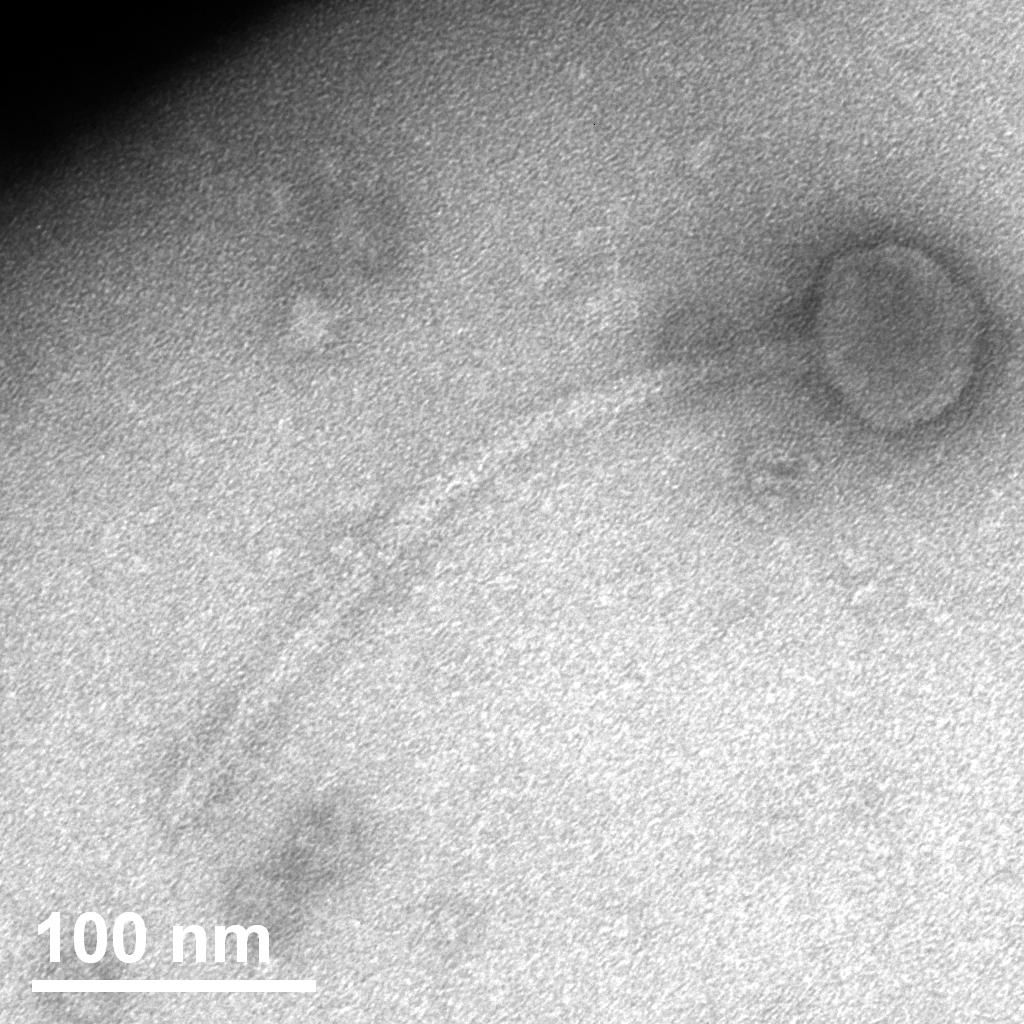
Soos was imaged using negative-stain transmission electron microscopy. Transmission Electron Microscopy (TEM) images show a siphovirus bacteriophage, with an average capsid diameter of 64.0 ± 0.9 nm and an average tail length of 273 ± 15 nm.

Soos, a novel bacteriophage that infects *Gordonia rubripertincta* NRRL B-16540*,* was isolated from a soil sample from Jeffersonville, Indiana, USA (Global Positioning System [GPS] coordinates 38.34446 N, 85.81798 W) using standard protocols (3, 4). The sample was washed in peptone yeast calcium agar (PYCa) media, which was then filtered through a 0.22 µm filter. *G. rubripertincta* was added to the filtrate and shaken at 250 rpm for 5 days at 26°C. An aliquot of enriched sample was filtered (0.22 µm), mixed with *G. rubripertincta,* and plated by soft-agar overlay. Incubation at 26°C for 2 days revealed 1 mm diameter, clear, circular plaques with cloudy halos. Soos was purified through two additional rounds of plating. Viral particles revealed siphovirus morphology (Fig. 1) with an average capsid diameter of 64.0 ± 0.9 nm and an average tail length of 273 ± 15 nm.

DNA was extracted from a plate lysate using the QIAGEN DNeasy Blood and Tissue Kit (5), prepared using New England Biolabs Ultra II Library Kit, and sequenced using Illumina MiSeq (v3 reagents). There were 17,286 150-base single-end reads providing 39× coverage. The raw reads were assembled with Newbler v2.9 and checked for completeness with Consed v29, using default settings (6). The genome is 57,509 base pairs in length with a guanine-cytosine (GC) content of 65.5%, and circularly permuted (6), as determined by comparison to similar phages with known ends and verified by the buildup of start reads using Consed v29.

The following programs were employed to annotate the genome as previously described (4) using default parameters: DNA Master (v5.23.6 Build 2705 24 October 2021) (https://phagesdb.org/DNAMaster/), Glimmer (v3.02b) (7), GeneMark [v2.5p (09.08.06)] (8), Starterator (v532; http://phages.wustl.edu/starterator/), Phamerator (Actino_Draft v532) (9), BLASTp (v2.14.0) (10), HHPred [utilizing databases: PDB_mmCIF70_10_Jan, Pfam-A_v35, UniProt-SwissProt-viral70_3_Nov_2021, and NCBI_Conserved_Domains(CD)_v3.19] (11), Aragorn (v1.2.41) (12), tRNAscanSE (v2.0.012) (13), and DeepTMHMM (version 1.0.24) (14). All 87 annotated genes were protein-coding genes transcribed in the same direction. No tRNA genes were identified. Twenty-eight genes were assigned putative functions, including three HNH endonucleases, an additional strand, catalytic E (ASCE) ATPase, an endolysin, and a holin. Five additional gene products were predicted to be membrane proteins. Only two genes (21 and 23) were found without homologs in the Actinobacteriophage database (2).

Actinobacteriophages with 35% gene content similarity (GCS) or higher are assigned to the same cluster (2, 15). Soos shares 84.1% GCS with annotated phage Clawz (GenBank accession MT498058), and 90.6% GCS with the draft genome of phage Sting (https://phagesdb.org/phages/Sting), the only other phages in cluster CP. Like Clawz, Soos includes a simple lysis cassette with a single, large endolysin gene preceding a holin gene (16). Nucleotide sequence comparison of Soos using BLASTn revealed that outside of cluster CP, Soos is most similar to GMA4 (GenBank accession NC_030939.1), a phage without sufficient GCS to be assigned to any cluster and with which Soos shares ~4% GCS.

## Data Availability

Soos GenBank accession OR475291.1, Sequence Read Archive (SRA) SRX20165784.
